# SF3b4: A Versatile Player in Eukaryotic Cells

**DOI:** 10.3389/fcell.2020.00014

**Published:** 2020-01-30

**Authors:** Feng Xiong, Sha Li

**Affiliations:** ^1^State Key Laboratory of Crop Biology, College of Life Sciences, Shandong Agricultural University, Tai’an, China; ^2^Department of Plant Biology and Ecology, College of Life Sciences, Nankai University, Tianjin, China

**Keywords:** spliceosome, Nager syndrome, tumorigenesis, transcription, cell signaling

## Abstract

Spliceosomes are large protein-RNA complexes regulating pre-mRNA processing in eukaryotes. *SF3b4* encodes a core subunit of the U2-type spliceosome, loss- or gain-of-function of which often associates with abnormal cell growth, leading to tumorigenesis. Homologs of *SF3b4* in other phyla are also essential. In this review, we summarize recent findings on the function of SF3b4. Importantly, we highlight the versatile roles of SF3b4, not only as a component for pre-mRNA splicing, but also as a regulator for transcription, translation, and cell signaling. Recent studies of SF3b4 homologs in different species across evolution will facilitate a better understanding of human diseases caused by the malfunction of SF3b4, such as Nager syndrome (NS) and cancer, in the future.

## Introduction

In eukaryotic cells, pre-mRNA splicing is an important step for gene expression with precise removal of introns from pre-mRNA to give rise to mature mRNA. Pre-mRNA splicing takes place in a large RNA-protein complex known as spliceosome (Matera and Wang, 2014). Spliceosome consists of a set of snRNPs, including the U1, U2, U4/U6, and U5 complexes. The U1 and U2 snRNPs recognize the 5′ splicing site and the branch point sequence of pre-mRNAs, respectively, and form a pre-spliceosome. The pre-spliceosome then associates with the pre-assembled U4/U6,U5 tri-snRNP to form spliceosome (Wan et al., 2019; Wilkinson et al., 2019).

*SF3b4* encodes a core subunit of the metazoan SF3b complex, which is a part of the U2-type spliceosome (Champion-Arnaud and Reed, 1994). In human, SF3b4 interacts with another spliceosome component SF3b145, and thus forms a protein complex that is thought to mediate the tethering of U2 snRNP to the branch site of pre-mRNAs (Champion-Arnaud and Reed, 1994; Gozani et al., 1996). Sequences and domain architectures of both SF3b4 and SF3b145 are well conserved among eukaryotes (Igel et al., 1998; Bernier et al., 2012; Devotta et al., 2016; Xiong et al., 2019).

Downregulating or depleting human *SF3b4* resulted in various diseases, such as tumorigenesis (Denu and Burkard, 2017; Zhou et al., 2017; Liu et al., 2018; Shen and Nam, 2018; Shen et al., 2018) and Nager syndrome (NS) that is characterized by defective craniofacial formation (Bernier et al., 2012). On the other hand, overexpressing *SF3b4* can also promote tumorigenesis in hepatocellular carcinoma (HCC) (Liu et al., 2018; Shen et al., 2018), suggesting an essential role of *SF3b4* in cell growth. Indeed, homologs of *SF3b4* in other phyla have also been proven to be essential (Igel et al., 1998; Bernier et al., 2012; Devotta et al., 2016; Xiong et al., 2019).

Despite the essential roles of *SF3b4* and its homologs in different species, molecular mechanisms underlying their function in cell growth and survival remain obscure. Recently, emerging evidences reveal that SF3b4 plays roles not only in pre-mRNA splicing but also in transcription, translation, as well as cell signaling. This review summarizes these recent advances on the function of SF3b4 and provides a systematic and perspective view of this versatile protein.

## Biological Function of SF3b4 and Its Homologs

**FIGURE 1:**
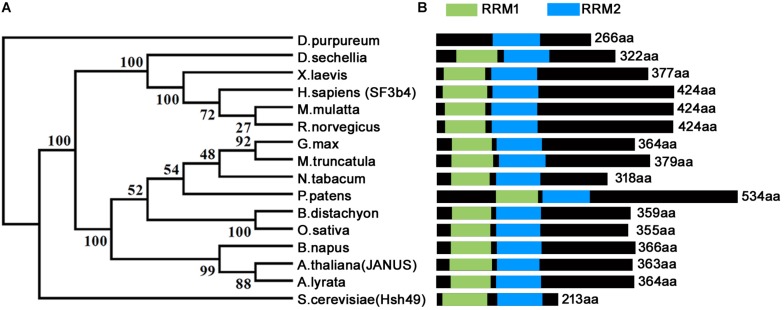
Phylogenetic analysis and schematic representation of domain architecture of SF3b4 protein homologs. **(A)** Phylogenetic analysis indicates that SF3b4 shares high similarity with putative homologous proteins from other organisms. Reproduced from Xiong et al. (2019) with permission. **(B)** SF3b4 and its homlogs contain two RRM domains.

Homologs of *SF3b4* have been proven to be conservative and essential for eukaryotes across different phyla (Figure 1) (Igel et al., 1998; Bernier et al., 2012; Devotta et al., 2016; Xiong et al., 2019). Functional loss of yeast *SF3b4* homolog, *Hsh49*, caused lethality (Igel et al., 1998). The same applies for *Arabidopsis thaliana*, the model plant species and a representative of multicellularity. Functional loss of *Arabidopsis JANUS*, an *SF3b4* homolog, results in embryo lethality (Xiong et al., 2019). *JANUS* is highly expressed in developing embryos from the zygotic stage to the cotyledon stage (Xiong et al., 2019). Consistent with its expression, functional loss of *JANUS* caused the disruption of cell division patterns (Xiong et al., 2019) that are strictly followed during *Arabidopsis* embryogenesis (Lau et al., 2012). Consequently, *JANUS* embryos were arrested at early stages, leading to lethality (Xiong et al., 2019).

Null mutants of human *SF3b4* was never obtained likely due to lethality (Marques et al., 2016). By studying the heterozygous *SF3b4* mutants, which are haploinsufficient (Bernier et al., 2012; Petit et al., 2014; Marques et al., 2016), it was determined that *SF3b4* is a causative factor for NS, an acrofacial dysostosis that causes upper limb and facial-mandibular defects (Wieczorek, 2013). Indeed, mice *SF3b4* is expressed with a high level in limbs and somites at embryonic day 11, when limbs started a rapid development (Wieczorek, 2013), consistent with its roles in skeletal development. In addition, downregulating *Xenopus SF3b4* in embryos led to a reduced number of neural crest progenitor cells and resulted in hypoplasia of neural crest-derived craniofacial cartilages, partially phenocoping the defects of skeletogenesis in NS patients (Devotta et al., 2016).

Recently, compelling evidences demonstrated the involvement of *SF3b4* in tumorigenesis. *SF3b4* is dramatically up-regulated in HCC compared to non-cancerous tissues (Xu et al., 2015; Iguchi et al., 2016). On the other hand, enhanced *SF3b4* expression is positively associated with the occurrence of intrahepatic metastasis and poor prognosis (Shen et al., 2018), suggesting that *SF3b4* is a potential oncogene in HCC. Indeed, *SF3b4* knockdown or depletion significantly inhibited the proliferation and metastasis of HCC cells both *in vitro* and *in vivo* (Shen et al., 2018). The elevated expression of *SF3b4* in HCC cells was attributed to a reduced level of *miRNA-133b*, which inhibits the expression of *SF3b4* (Liu et al., 2018). Consistently, the expression of *miRNA-133b* mimics the partly suppressed ability of *SF3b4*-overexpressing HCC cells to proliferate and migrate (Liu et al., 2018). In view of its importance in HCC tumorigenesis, *SF3b4* was thus suggested to be used as an early-stage diagnostic marker of HCC (Shen and Nam, 2018).

In contrast to the studies in HCC cells, which indicate *SF3b4* being an oncogene, the roles of SF3b4 in other tumorigenesis processes is complex. The expression of *SF3b4* was significantly decreased in pancreatic cancer cells. Further studies showed that overexpression of *SF3b4* in pancreatic cancer cells inhibited cell growth and motility, while suppressing *SF3b4* expression promoted the proliferation and migration of pancreatic cancer cells (Zhou et al., 2017). The distinct roles of *SF3b4* in different cancer cells need further investigation. However, these studies all point to the importance of a finely regulated level of *SF3b4* for metazoan cells.

## Molecular Function of SF3b4

### Splicing

**FIGURE 2:**
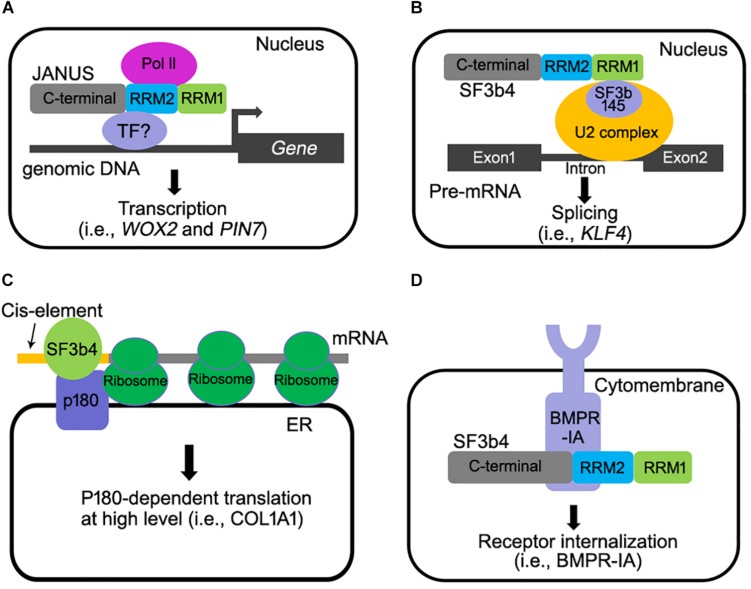
Multiple molecular functions of SF3b4 in eukaryotes. **(A)** *Arabidopsis* JANUS directly interacts with RNA polymerase II via its RRM2 domain and regulates Pol ll-mediated transcription of specific target genes (Xiong et al., 2019). **(B)** SF3b4 can interact with SF3b145 via its RRM1 domain and tether the U2 snRNP to the branch site in pre-mRNA splicing (Champion-Arnaud and Reed, 1994; Igel et al., 1998; van Roon et al., 2017; Shen and Nam, 2018). **(C)** SF3b4 interacts with p180 at ER and mediates translational control of secretory proteins (Ueno et al., 2019). **(D)** SF3b4 interacts with BMPR-IA receptor kinase at cell membrane and facilitates its internalization. Both the RRM2 and C-terminal motif of SF3b4 are required for the interaction (Pignatti et al., 2014).

As a component of the spliceosome, SF3b4 participates in RNA splicing (Figure 2B) (Igel et al., 1998). Studies in metazoans and yeast showed that downregulating *SF3b4* inhibited RNA splicing (Champion-Arnaud and Reed, 1994; Igel et al., 1998). In yeast, repression of *Hsh49* expression resulted in the accumulation of unspliced U3 RNA, indicating that efficient splicing *in vivo* requires *Hsh49* expression (Igel et al., 1998). In humans, overexpressing *SF3b4* resulted in a mis-splicing of Kruppel-like factor 4 (*KLF4*), a tumor suppressor-encoding gene, into a non-functional transcript in cancer cells, and thus promoting tumorigenesis in HCC (Shen et al., 2018). SF3b4 and its homologs contain two RNA-recognition motifs (RRM1 and RRM2). The RRM1 domain of SF3b4 is required for its interaction with SF3b145, the second large subunit of the SF3b complex, and for U2 snRNA binding (Igel et al., 1998; Kuwasako et al., 2017; van Roon et al., 2017). The crystal structure of yeast spliceosome reveals a similar role of Hsh49-RRM1 (van Roon et al., 2017). The α-helical surface of Hsh49-RRM1, as opposite to the four-stranded β-sheet, interacts with Cus1, leaving the canonical RNA-binding surface of Hsh49-RRM1 available to bind snRNA (van Roon et al., 2017). At the same time, the binding of Hsh49-RRM1 to the 5′ end region of U2 snRNA is enhanced by complexing with Cus1 (van Roon et al., 2017). Unlike the RRM1 domain whose roles have been clearly defined, the function of Hsh49-RRM2 is obscure, although the RRM2 domain of human SF3b4 was proposed to mediate pre-mRNA-binding (Tanaka et al., 1997). Substituting two amino acids within either Hsh49-RRM1 or Hsh49-RRM2, i.e., Hsh49^Y52D/F54D^ or Hsh49^C150D/Y152D^, resulted in lethality (Igel et al., 1998), suggesting that both RRM domains are critical for the function of Hsh49.

### Transcription

In *Xenopus* embryos, a depletion of *SF3b4* disrupted the formation of neural crest progenitors (Devotta et al., 2016). Surprisingly, pre-mRNA processing of a subset of transcriptional regulators that are critical for neural crest formation was not altered (Devotta et al., 2016). Rather, the expression of these genes was significantly reduced, even to an undetectable level in *SF3b4*-depleted embryos (Devotta et al., 2016). These results hinted at the possibility that *SF3b4* regulates transcription, directly or indirectly.

A study on Rodriguez syndrome, a severe form of NS, hinted at a similar transcriptional regulatory role of SF3b4 in humans (Marques et al., 2016). By RNA-seq analysis, it was shown that genes of the growth plate chondrocytes showed a significant reduction in expression levels whereas only slight changes in regards to their splicing was highlighted (Marques et al., 2016).

Studies in both human and *Xenopus* did not provide evidence supporting a direct involvement of SF3b4 in transcription regulation. However, a recent study in *Arabidopsis* convincingly showed that SF3b4 mediates the transcription of certain genes by recruiting RNA polymerase II complex (Pol II). *Arabidopsis JANUS* was initially identified as it is essential for embryogenesis (Xiong et al., 2019). Genetic, cellular, and molecular approaches were used to demonstrate that JANUS mediates embryo development through the transcription but not through RNA splicing of *WUSCHEL RELATED HOMEOBOX2* (*WOX2*) and *PIN-FORMED7* (*PIN7*), two genes critical for early embryonic pattern formation. JANUS contains two RRMs (RRM1 and RRM2), like their yeast and metazoan counterparts. Interestingly, this study reported that JANUS-RRM2 interacts with Pol II and such an interaction is critical for the transcription of *WOX2* and *PIN7* during early embryo development (Figure 2A).

The study in *Arabidopsis* convincingly demonstrates a direct role of SF3b4 in transcriptional regulation, which is independent from its role of being a component in the spliceosome. Given that the involvement of SF3b4 in transcription has been reported in metazoans and that SF3b4 is highly conserved during evolution, it is likely that similar mechanisms also apply for metazoan SF3b4. In fact, mice SF3b4 did interact with Pol II subunits through its RRM2 domain (Xiong et al., 2019).

### Translation

SF3b4 participates in translational control in YA7 cells (Figure 2C). p180 is an essential factor for high-rate biosynthesis of collagens and fibronectins on endoplasmic reticulum (ER) (Tanaka et al., 1997). It has been recently reported that SF3b4 interacts with p180 (Ueno et al., 2019). As a cofactor of p180, a co-expression of SF3b4 with p180 enhanced the association of mRNAs with the ER membrane and the assembly of polyribosomes for certain mRNAs (Ueno et al., 2019), leading to a high-rate biosynthesis of secreted proteins (Ueno et al., 2019). An interesting scenario worthy of further investigation is that a reduced SF3b4 level causes impaired collagen secretion, leading to NS (Ueno et al., 2019). Interestingly, the ER-mediated translational control of SF3b4 is cell-type specific (Ueno et al., 2019), suggesting that different cell types utilize SF3b4 through distinct mechanisms. Furthermore, a 5′UTR *cis*-element comprising the motif GAG-(X)3-ACA/G/C is required for both the ER association of SF3b4 and enhanced biosynthesis in a p180-dependent manner (Ueno et al., 2019). Although these findings appear to suggest a pivotal role for interaction between p180 with SF3b4 in translation control, it remains to be elucidated whether or not the specific interaction of p180with SF3b4 increases ribosome loading to specific mRNAs containing such a *cis*-element (Ueno et al., 2019).

### Cell Signaling

SF3b4 and its homologs are mostly nuclear localized (Champion-Arnaud and Reed, 1994; Watanabe et al., 2007; Xiong et al., 2019), which is consistent with their roles for pre-mRNA splicing and transcription. However, a portion of mice SF3b4 is also associated with the cell membrane together with Bone Morphogenetic Protein Receptor IA (BMPR-IA), a receptor kinase that regulates craniofacial development and cell-fate determination in embryos (Nishanian and Waldman, 2004; Bonilla-Claudio et al., 2012; Pignatti et al., 2014). Mice SF3b4 interacted with BMPR-IA and down-regulated the level of BMPR-IA at cell surface level (Figure 2D) (Nishanian and Waldman, 2004; Watanabe et al., 2007). These results suggested that SF3b4 somehow affects cell signaling through mediating receptor internalization. However, BMP signaling was not affected by the depletion of *SF3b4* in *Xenopus* embryos (Devotta et al., 2016), likely because the interaction between SF3b4 and BMPR-1A is cell-type dependent. In any case, the physiological significance of SF3B4-BMPR1A interaction remains to be investigated *in vivo*. In addition to BMRP-IA, the transcription factor STAT3 that is critical for apoptosis (Zhou et al., 2017) is regulated by SF3b4 as SF3b4 inhibits the phosphorylation of STAT3. This negatively regulates the signaling of STAT3 in pancreatic cancer cells (Zhou et al., 2017). How SF3b4 regulates the phosphorylation of STAT3 needs to be further investigated.

## Conclusion

Recent studies of SF3b4 in different species across evolution (Watanabe et al., 2007; Ueno et al., 2019; Xiong et al., 2019) have revealed the multiple roles that SF3b4 plays in transcription, in translation, in cell signaling in addition to the traditional pre-mRNA splicing. Results obtained from these studies will facilitate a better understanding of human diseases caused by the malfunction of SF3b4, such as NS, HCC, and pancreatic cancer in the future.

## Perspectives

Although *Arabidopsis* JANUS recruits Pol II for the transcription of *WOX2* and *PIN7* (Xiong et al., 2019), there is no recognizable DNA binding domains in JANUS. The method, in which, JANUS recruits Pol II specifically on its targets is still a mystery. There are multi-subunit complexes known as mediator bridge transcription regulators that bind with Pol II at specific *cis*-elements for the transcription initiation in eukaryotic cells (Soutourina et al., 2011; Dolan and Chapple, 2017). Whether JANUS fulfils the role of mediator whilst, recruiting DNA-binding transcription factors together with Pol II to the promoter regions of its target gene, is an interesting study that is worthy to be investigated further.

The confirmation of the direct role that SF3b4 plays in transcription is of great significance as it sheds a new light on the involvement of SF3b4 in human diseases and in tumorigenesis. However, as transcription and pre-mRNA processing are intimately coupled in time and in space (Bentley, 1999), it is challenging to separate the two functions of SF3b4. Structural biology may provide a solution to this problem. The crystal structures of Hsh49 have already been resolved and the key amino acids within Hsh49 for its interaction with other subunits of the spliceosome have been identified (van Roon et al., 2017). Mutations of these key residues can theoretically abolish the interaction of Hsh49 with the spliceosome without affecting its function in Pol II recruitment. In addition, identifying the direct transcription or splicing targets of SF3B4 is another angle in which this powerful protein can be understood in full.

The cell-type-specific role of SF3b4 in translational control is also intriguing (Ueno et al., 2019). From such an angle, the reduced SF3b4 levels in heterozygous *sf3b4* individuals might have resulted in impaired collagen secretion, leading to NS, therefore making this an interesting topic worthy to be investigated further. Analyses of collagens in fibroblasts from these patients will provide a vital clue for elucidating the mechanisms of skeletal malformation (Ueno et al., 2019).

In sum, the study of human *SF3b4*, an essential gene for our viability and health, will benefit substantially from studies of its yeast, metazoan, and even plant homologs, in which genetic manipulation is much faster and more convenient. A deeper understanding of SF3b4 and its evolutionary homologs may provide important clues to cue the related human diseases.

## Author Contributions

FX drafted the initial design. SL secured funding. Both authors contributed to the contents and approved the final version of the manuscript.

## Conflict of Interest

The authors declare that the research was conducted in the absence of any commercial or financial relationships that could be construed as a potential conflict of interest.
